# Macrophage Inhibitory Factor in Myocardial Oxidative Stress and Inflammation During Thioacetamide-Induced Liver Fibrosis: Modulation by Betaine

**DOI:** 10.3390/cimb47090728

**Published:** 2025-09-09

**Authors:** Jasmina Djuretić, Jelena Filipovic, Milica Brankovic, Sanja Stankovic, Janko Samardzic, Danijela Vucevic, Tatjana Radosavljevic

**Affiliations:** 1 Department of Pathobiology, Faculty of Pharmacy, University of Belgrade, 11000 Belgrade, Serbia; jasmina.djuretic@pharmacy.bg.ac.rs; 2 Institute of Pathology, Faculty of Medicine, University of Belgrade, 11000 Belgrade, Serbia; vjesticaj@gmail.com; 3 Institute of Pharmacology, Clinical Pharmacology and Toxicology, Faculty of Medicine, University of Belgrade, 11000 Belgrade, Serbia; milicabrankovic137@yahoo.com (M.B.);; 4 Centre for Medical Biochemistry, University Clinical Centre of Serbia, 11000 Belgrade, Serbia; sanjast2013@gmail.com; 5 Department of Physiology, Faculty of Medical Sciences, University of Kragujevac, Svetozara Markovica 69, 34000 Kragujevac, Serbia; 6 Institute of Pathophysiology “Ljubodrag Buba Mihailovic”, Faculty of Medicine, University of Belgrade, 11000 Belgrade, Serbia; danijela.vucevic@med.bg.ac.rs

**Keywords:** liver fibrosis, thioacetamide, macrophage inhibitory factor, heart, oxidative stress, inflammation, betaine, mice

## Abstract

Chronic liver disease is closely associated with impaired cardiovascular function. Cardiac dysfunction is caused in part by oxidative stress and increased levels of proinflammatory and profibrogenic mediators in myocardial tissue. The present study aims to investigate the role of betaine in the modulation of MIF-mediated oxidative stress, inflammation, and fibrogenesis in heart during TAA-induced liver fibrosis in mice. The experiment is performed on wild-type and knockout *MIF^−/−^* C57BL/6 mice (MIF^−/−^ group). They are randomly divided into groups: Control; Bet-group, received betaine (2% *wt*/*v* dissolved in drinking water); MIF^−/−^ mice group; MIF^−/−^+Bet; TAA-group, treated with TAA (200 mg/kg b.w.), intraperitoneally, 3×/week/8 weeks); TAA+Bet; MIF^−/−^+TAA, and MIF^−/−^+TAA+Bet group. After eight weeks of treatment, animals are sacrificed and heart samples are taken to determine oxidative stress parameters, proinflammatory cytokines, profibrogenic factors, and histopathology of myocardial tissue. Our results suggest that MIF contributes significantly to lipid peroxidation of cardiomyocytes, as well as oxidative and nitrosative stress in myocardial tissue in mice with TAA-induced liver fibrosis compared to the control group. In addition, MIF was important for myocardial expression of the proinflammatory cytokines IL-6 and TNF as well as the profibrogenic mediators TGF-β1 and PDGF-BB in TAA-treated mice. Notably, betaine attenuated MIF effects in myocardial tissue reducing levels of MDA, AOPP, TNF, TGF-β1, PDGF-BB and increasing SOD and catalase activity in the coexistence of liver fibrosis. These results emphasize the potential of betaine as a therapeutic agent in mitigating MIF effects and demonstrate the need for further research into its optimal dosage and efficacy in preventing or slowing down cardiac dysfunction in patients with liver cirrhosis.

## 1. Introduction

Liver diseases are widespread worldwide and cause considerable morbidity and mortality [1]. The incidence and prevalence of chronic liver disease (CLD) and its progression to fibrosis, cirrhosis and hepatocellular carcinoma (HCC) are very high [1]. Liver fibrosis is the result of chronic damage to the liver caused by certain etiological factors such as chronic alcohol consumption, obesity, metabolic syndrome, viral infections and the accumulation of drugs/toxins. The consumption of or exposure to chemicals poses a serious health risk. Many organs can be damaged in this way, including the liver. Chemicals that can cause direct liver damage are found in nature or are products of the chemical or pharmaceutical industry [2]. Fibrosis is an adaptive response to prolonged liver damage and is characterized by excessive accumulation of extracellular matrix (ECM) proteins, which is characteristic of most types of chronic liver disease [3,4]. Therefore, the main goal of preventive and therapeutic strategies is to mitigate the progression of the disease from steatosis to fibrosis, cirrhosis and HCC and their complications. In this context, the importance of numerous experimental models of CLD should be emphasized, which provide a good basis for studying the mechanisms of fibrosis progression and testing antifibrogenic agents, thus further contributing to the prevention and treatment of liver disease and complications of cirrhosis [5,6].

Thioacetamide (TAA, IUPAC: ethanethioamide, C_2_H_5_NS) [7] is a sulfur-containing organic compound that is widely used in animal studies as a hepatotoxin, nephrotoxin and carcinogen [8]. Thioacetamide (TAA)-induced hepatotoxicity is a suitable animal model for acute and chronic liver injury [5,6]. Chronic administration of TAA leads to altered redox homeostasis, inflammation, hepatocyte necrosis and liver fibrosis [8,9,10,11]. In addition to hepatotoxicity, TAA has been confirmed to exert toxic effects on other organs [8,12,13,14,15]. In the liver, TAA is metabolized via cytochrome P450 to highly reactive TAA-S-oxide and TAA-S-S-dioxide, which leads to the triggering of oxidative stress, inflammation and fibrogenesis [8,9].

However, the role of oxidative stress, inflammation and fibrogenic mediators in myocardial damage during liver fibrosis/cirrhosis is not yet sufficiently understood. The results of some studies show that oxidative stress and inflammation may be involved in cardiac dysfunction in liver cirrhosis [16,17]. This is very important as cardiac changes in later stages of cirrhosis in humans could be the cause of the development of ascites, hepatopulmonary syndrome and hepatorenal syndrome [18]. The results of the study by Fattouh et al. also show that children with liver cirrhosis exhibit diastolic dysfunction of the heart [19]. Indices of liver fibrosis derived from T1 mapping correlate with cardiac parameters such as left ventricular hypertrophy, left atrium dysfunction and myocardial fibrosis [20].

Macrophage migration inhibitory factor (MIF) is a multifunctional cytokine that is released by immune cells and contributes to the regulation of the immune system [21,22]. MIF is an important proinflammatory cytokine that exhibits both cytokine and chemotactic properties and regulates the innate immune response [22,23,24,25,26]. MIF is released by various types of immune cells, endothelial cells, tissue macrophages, some parenchymal and endocrine cells [24,26]. This factor stimulates the synthesis of numerous proinflammatory mediators such as TNF, IL-6, IL-1 and others [21,23,25]. MIF is a key cytokine in chronic liver disease [9,23,24,25], sepsis [26], autoimmune diseases [27], asthma [28] and other diseases [29,30]. MIF is a master regulator of inflammatory cardiovascular disorders [31,32,33,34]. This factor has a dual function. Intracellular MIF has a cardioprotective effect in the early stages of myocardial ischemia/reperfusion injury, as demonstrated in studies [35,36]. On the other hand, high levels of MIF in circulation have been associated with promoting the progression of atherosclerosis as a proinflammatory cytokine [34,37]. However, its ability to reduce apoptosis in the myocardium during reperfusion emphasizes its potential as an intracellular thiol oxidoreductase [35,36].

Betaine (trimethylglycine) is found in plants, animals, microorganisms and foods such as wheat germ, bran, vegetables and seafood [38,39,40]. As an oxidative metabolite of choline, it acts as an osmolyte and methyl donor in the methionine-homocysteine cycle and has antioxidant properties by increasing the content of S-adenosylmethionine and methionine [38,39,41]. Studies have shown its hepatoprotective effect in various experimental models [9,12,42,43,44]. In addition, betaine has neuroprotective properties, protects cardiac function and inhibits pancreatic steatosis [38]. Betaine also reduces oxidative stress, endoplasmic reticulum stress, inflammation and the development of cancer.

It is well known that chronic liver disease and heart failure often coexist [45], i.e., cardiac dysfunction is an important complication of liver cirrhosis, but the role of MIF in oxidative/inflammatory myocardial damage during liver cirrhosis is not clear enough. Therefore, the aim of our study is to investigate the role of MIF in oxidative stress and inflammation in the myocardium during TAA-induced liver fibrosis. Considering the importance of antioxidants in the preventive and therapeutic strategy of chronic diseases, this study also investigates the modulating effect of betaine on myocardial MIF-mediated oxidative stress and inflammation in TAA-induced liver fibrosis.

## 2. Materials and Methods

### 2.1. Animals

Two types of animals were used in this study: male C57BL/6 wild-type mice and C57BL/6 mice with MIF knockout (MIF^−/−^), 8 weeks old, weighing 21–25 g, reared at the Military Medical Academy in Belgrade. The animals were kept under standard laboratory conditions (temperature 22 ± 2 °C, relative humidity 50 ± 10%, 12/12 light/dark cycle with lights switched on at 9.00 am). They were housed in cages where they had free access to water and standard chow diet during the experiment. All experimental procedures were in accordance with the Directive of the European Parliament and of the Council (2010/63EU) and were approved by the Ethical Committee of the University of Belgrade (Permission No. 6600/2, approved on 14 October 2019).

### 2.2. Experimental Design

A total of 56 animals were used in this experiment. Before the experiment, all 56 mice were fed a control diet. At 8 weeks of age, they were randomly divided into 8 groups (n = 7/per group): 1. control group (C), fed with standard chow diet; 2. Bet group, fed with standard chow diet and supplemented with betaine; 3. MIF^−/−^ group, fed with standard chow diet; 4. MIF^−/−^+Bet group, fed with standard chow diet and supplemented with betaine; 5. TAA group fed with standard chow diet and treated with TAA; 6. TAA+Bet group treated with TAA and supplemented with betaine; 7. MIF^−/−^+TAA group fed with standard chow diet and treated with TAA; 8. MIF^−/−^+TAA+Bet group fed with standard chow diet and treated with TAA and supplemented with betaine. Liver injury was induced by TAA (200 mg/kg) dissolved in 200 mL PBS, administered intraperitoneally three times a week over a period of 8 weeks. The solution was freshly prepared each week and stored at 4 °C. TAA was obtained from Sigma (St. Louis, MO, USA). At the same time, the control group (C), the Bet group, the MIF^−/−^ group and the MIF^−/−^+Bet group received vehicle (saline 0.9% NaCl) in the same manner. Betaine (purchased from Biomedicals) was administered to the animals dissolved in drinking water (2% *w*/*v*) [46,47]. Each animal was housed in a separate cage with free access to drinking fluid. The betaine solution was the only source of drinking fluid provided to the mice, and its intake was carefully monitored daily. The day before sacrifice, the mice fasted overnight and weighed before sacrifice (Table S1). Sacrifice was performed by exsanguination under ketamine anesthesia. Ketamine (100 mg/kg) was administered intraperitoneally. Heart samples were taken to determine the proinflammatory citokines: interleukin (IL)-6 and tumor necrosis factor (TNF) as well as profibrogenic factors: platelet-derived growth factor (PGDF) and transforming growth factor (TGF)-β1.

### 2.3. Preparation of the Myocardial Samples and Biochemical Analysis

Myocardial samples for biochemical analysis were homogenized on ice in cold buffered 0.25 M sucrose medium (Serva, New York, NY, USA, Heidelberg, Germany), 10 mM phosphate buffer (pH = 7.0) and 1 nM ethylenediaminetetraacetic acid (EDTA; Sigma Chem. Co. St. Louis, MO, USA). The homogenates were centrifuged at 2000× *g* for 15 min at 4 °C. The crude sediments were dissolved in a sucrose medium and centrifuged. The supernatant was transferred to tubes and centrifuged at 3200× *g* for 30 min at 4 °C. The resulting sediments were dissolved in deionized water and after one hour of incubation the samples were centrifuged at 3000× *g* for 15 min at °C and the supernatants were stored at −70 °C. The proteins were determined using the Lowry method with bovine serum albumin as standard [48]. ELISA kits from BD Bioscience (San Diego, CA, USA) were used to determine the concentration of cytokines and profibrogenic factors. All these parameters were determined according to the manufacturer’s instructions.

### 2.4. Determination of Oxidative/Nitosative and Antioxidative Parameters in Myocardial Tissue

Lipid peroxidation was assessed by measuring malondialdehyde (MDA) levels using a spectrophotometric method based on its reaction with thiobarbituric acid, as described by Girotti et al. [49]. Results were reported in nanomoles of MDA per milligram of protein (nmol/mg protein).

The spectrophotometric assessment of advanced oxidation protein products (AOPP) was carried out according to the method described by Witko et al. [50]. To prepare the samples, 200 µL of serum was diluted 1:5 in PBS. Subsequently, 10 µL of 1.16 M potassium iodide and 20 µL of acetic acid were added to each tube. The absorbance of the resulting mixture was measured immediately at 340 nm, using a blank composed of 1000 µL of PBS, 10 µL of potassium iodide, and 20 µL of acetic acid. A chloramine T solution (0–100 µmol/L) served as the standard, with a linear absorbance response at 340 nm within this range. AOPP levels were expressed as micromoles per liter (µmol/L) of chloramine T equivalents [51].

Nitrite (NO_2_^−^) levels in liver tissue were measured using the Griess reagent method, in accordance with established protocols [52].

Total superoxide dismutase (SOD; EC 1.15.1.1) activity in liver tissue was determined spectrophotometrically by measuring the inhibition of epinephrine autooxidation at 480 nm. The reaction was initiated by adding 10 mM epinephrine (Sigma, St. Louis, MO, USA) to a sodium carbonate buffer (50 mM, pH 10.2; Serva, Feinbiochemica, Heidelberg, New York, NY, USA) containing 0.1 mM EDTA (Sigma, St. Louis, MO, USA) [53].

Catalase (CAT) activity in liver tissue was measured spectrophotometrically by monitoring the decrease in hydrogen peroxide absorbance at 240 nm and expressed as units of activity per milligram of protein [54]. One unit of catalase activity was defined as the amount of enzyme that decomposes 1.0 mmol of hydrogen peroxide per minute at pH 7.0 and 25 °C.

Total thiol content was determined using a spectrophotometric assay based on 2,2-dithiobis nitrobenzoic acid (DTNB, also known as Ellman’s reagent) [55]. A serum aliquot was combined with Tris-EDTA buffer, followed by the addition of DTNB. After a 15 min incubation at room temperature, absorbance was recorded at 405 nm. A reagent blank (without sample) and a sample blank (with methanol replacing DTNB) were prepared similarly. A reduced glutathione (GSH) solution ranging from 50 to 100 μmol/L served as a standard, and total thiol concentrations were expressed in μmol/L.

### 2.5. Determination of Proinflammatory Cytokines (IL-6 and TNF) and Profibrogenic Mediators (TGF-b1 and PDGF-BB) in Myocardial Tissue

For the determination of cytokines and profibrogenic factors, myocardial tissues (50 mg) were homogenized in 10 volumes of ice-cold PBS containing protease inhibitors. The homogenates were centrifuged at 12,000× *g* for 15 min at 4 °C, and supernatants were collected and stored at −80 °C. The concentrations of proinflammatory cytokines (IL-6 and TNF) in the supernatants were determined using specific ELISA kits from BD Bioscience (San Diego, CA, USA). For the quantification of profibrogenic mediators (TGF-β1 and PDGF-BB), ELISA kits from Elabscience (Houston, TX, USA) were used, following the manufacturer’s instructions. All values were normalized to the weight of myocardial tissue.

### 2.6. Preparation of Myocardial Tissue for Pathohistological Analysis 

The myocardial tissue was sectioned and incubated in 10% formalin solution at room temperature. After fixation, the myocardial samples were processed according to the standard method. The tissue was mounted in 4 cm thick paraffin sections and then stained with haematoxylin and eosin (H&E) and Masson-Trichrome (MT) to assess myocardial damage histologically. Slides were analyzed and photographed using a combined photobinocular light microscope (model BX53; Olympus, Hamburg, Germany) equipped with a DP12 CCD camera (Olympus, Hamburg, Germany) to characterize the histopathological changes at 400× magnification. All samples were assessed by an experienced pathohistologist who was blinded to the experiment. The following parameters were selected to indicate the severity of morphological damage to the myocardium after administration of TAA and betaine: hypereosinophilic cytoplasm, loss of striation, nuclear loss, necrosis, inflammatory infiltrate, interstitial fibrosis and vascular changes.

### 2.7. Statistical Analysis

Data were presented as means ± SD and analyzed using GraphPad Prism 9.5.1 software. As the normal distribution of parameters was confirmed by the Kolmogorov–Smirnov test, one-way analysis of variance (ANOVA) with Tukey’s post hoc test was used for testing the difference among groups. Statistical significance was set at *p* < 0.05.

## 3. Results

### 3.1. Oxidative/Nitrosative and Antixidative Parameters in Myocardial Tissue

There were no significant differences in the oxidative/nitrosative and antioxidative parameters in the myocardial tissue between the MIF^−/−^ group and the wild-type mice fed the standard diet.

**Figure 1:**
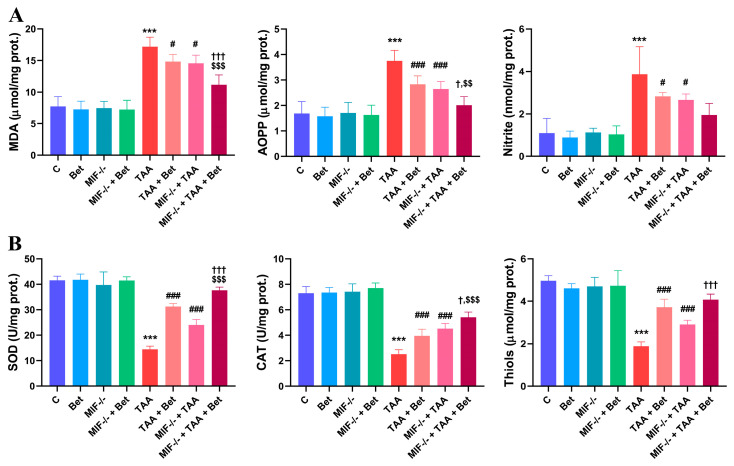
Effects of MIF and betain on the oxidative/nitrosative (**A**) and antioxidative (**B**) parameters in myocardial tissue of mice treated with TAA. The data are presented as mean ± SD from 7 mice per group. Significance of the difference was estimated by using one-way analysis of variance (ANOVA) with Tukey’s post hoc test; *** *p* < 0.001 vs. C; ^#^ *p* < 0.05, ^###^ *p* < 0.001 vs. TAA; ^†^ *p* < 0.05,^†††^ *p* < 0.001 vs. MIF^−/−^+TAA; ^$$^ *p* < 0.01, ^$$$^ *p* < 0.001 vs. TAA+Bet.

The myocardial MDA concentration was significantly increased (*p* < 0.001) in the TAA group (17.20 ± 1.49 μmol/mg prot.) compared to the control group (7.73 ± 1.56 μmol/mg prot.). The MDA concentration in the myocardial tissue decreased significantly in the TAA+Bet (14.83 ± 1.13 mmol/mg) and MIF^−/−^+TAA group (14.55 ± 1.29 μmol/mg prot.) compared to the TAA group (*p* < 0.05). In addition, the MDA concentration in the myocardial tissue was significantly lower in MIF^−/−^+TAA+Bet (11.15 ± 1.54 μmol/mg prot.) compared to MIF^−/−^+TAA and TAA+Bet (*p* < 0.001) (Figure 1A).

The content of advanced oxidation protein products (AOPPs) in myocardial tissue was significantly increased (*p* < 0.001) in the TAA group (3.75 ± 0.41 μmol/mg prot.) compared to the control group (1.69 ± 0.46 μmol/mg prot.). The myocardial AOPPs concentration decreased (*p* < 0.001) in the TAA+Bet (2.83 ± 0.33 mmol/mg) and MIF^−/−^+TAA group (2.65 ± 0.29 μmol/mg prot.) compared to the TAA group. In addition, the level of myocardial AOPPs was significantly decreased in MIF^−/−^+TAA+Bet (2.01 ± 0.34 μmol/mg prot.) compared to MIF^−/−^+TAA and TAA+Bet (*p* < 0.05 and *p* < 0.01, respectively) (Figure 1A).

The concentration of cardiac nitrite (NO_2_^−^) was significantly increased in the TAA group (3.86 ±1.31 nmol/mg protein) compared to the control group (1.09 ± 0.70 nmol/mg protein) (*p* < 0.001). Cardiac nitrite concentration was decreased in the TAA+Bet (2.83 ± 0.18 nmol/mg) and MIF^−/−^+TAA group (2.65 ± 0.29 μmol/mg prot.) compared to the TAA group (*p* < 0.05). In addition, the cardiac nitrite content was slightly reduced in the MIF^−/−^+TAA+Bet group (1.95 ± 0.54 nmol/mg prot.) compared to the TAA+Bet and MIF^−/−^+TAA groups, but this was not statistically significant (Figure 1A).

Treatment with TAA significantly (*p* < 0.001) decreased total myocardial SOD activity in the TAA group (14.40 ± 1.26 U/mg prot.) compared to the control group (41.50 ± 1.64 U/mg prot.). On the other hand, treatment with betaine led to a significant increase (*p* < 0.001) in SOD activity in the TAA+Bet (31.29 ± 1.12 U/mg prot.) compared to the TAA group. Furthermore, a significant increase (*p* < 0.001) in the activity of these enzymes was observed in the MIF^−/−^+TAA+Bet group (37.66 ± 1.22 U/mg prot.) compared to the MIF^−/−^+TAA group (23.96 ± 2.28 U/mg prot.) and the TAA+Bet group (Figure 1B).

Further analysis of cardiac antioxidant capacity revealed that CAT activity was significantly decreased (*p* < 0.001) in the TAA group (2.50 ± 0.35 U/mg prot.) compared to control values (7.30 ± 0.53 U/mg prot.). CAT activity was significantly increased in the TAA+Bet (3.95 ± 0.51 U/mg prot.) and MIF^−/−^+TAA (4.51 ± 0.41 U/mg prot.) groups compared to the TAA group (*p* < 0.001). Moreover, CAT activity was significantly increased in the MIF^−/−^+TAA+Bet group (5.41 ± 0.41 U/mg prot.) compared to the MIF^−/−^+TAA group (4.51 ± 0.41 U/mg prot.) and the TAA+Bet group (3.95 ± 0.51 U/mg prot.) (*p* < 0.05 and *p* < 0.001, respectively) (Figure 1B).

The thiol concentration in the myocardium was significantly lower (*p* < 0.001) in the TAA group (1.88 ± 0.21 μmol/mg prot.) compared to the control group (4.96 ± 0.24 μmol/mg prot.). In contrast, the cardiac thiol concentration was significantly higher (*p* < 0.001) in the TAA+Bet (3.72 ± 0.37 μmol/mg prot.) and MIF^−/−^+TAA group (2.90 ± 0.21 μmol/mg prot.) compared to the TAA group (1.88 ± 0.21 μmol/mg prot.). In addition, the cardiac thiol content was significantly increased in the MIF^−/−^+TAA+Bet group (4.07 ± 0.27 μmol/mg prot.) compared to the MIF^−/−^+TAA group (*p* < 0.001). On the other hand, the thiol concentration in the myocardium of the MIF^−/−^+TAA+Bet group was increased compared to the TAA+Bet group, but not significantly (Figure 1B).

### 3.2. Determination of Proinflammatory Cytokines (IL-6 and TNF) in Myocardial Tissue

**Figure 2:**
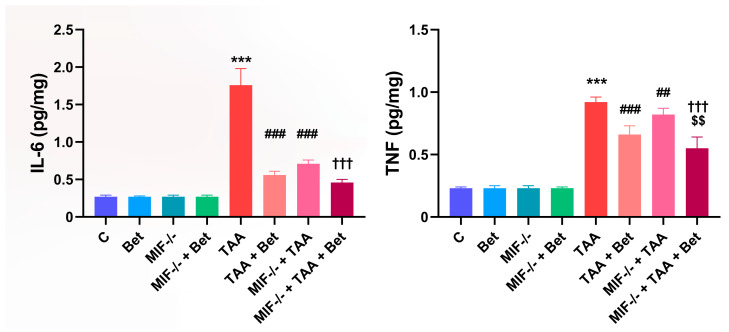
Effects of MIF and betain on the levels of proinflammatory cytokines (IL-6 and TNF) in myocardial tissue of mice treated with TAA. The data are presented as mean ± SD from 7 mice per group. Values were normalized to the weight of myocardial tissue. Significance of the difference was estimated by using one-way analysis of variance (ANOVA) with Tukey’s post hoc test; *** *p* < 0.001 vs. C; ^##^ *p* < 0.01, ^###^ *p* < 0.001 vs. TAA; ^†††^ *p* < 0.001 vs. MIF^−/−^+TAA; ^$$^ *p* < 0.01 vs. TAA+Bet.

There were no significant differences in the concentration of IL-6 and TNF in the myocardial tissue between the MIF^−/−^ group and the wild-type mice fed the standard diet (Figure 2). Treatment with TAA led to a significant increase in IL-6 concentration in the myocardial tissue of the TAA group (1.76 ± 0.22 pg/mg) compared to the control group (0.27 ± 0.02 pg/mg) (*p* < 0.001). The myocardial IL-6 concentration in the TAA+Bet (0.56 ± 0.05 pg/mg) and MIF^−/−^+TAA (0.71 ± 0.05 pg/mg) groups was significantly reduced compared to the TAA group (*p* < 0.001). Similarly, a decrease in myocardial IL-6 concentration was observed in the MIF^−/−^+TAA+Bet group (0.46 ± 0.04 pg/mg) compared to the TAA+Bet and MIF^−/−^+TAA groups, but this decrease was only significant compared to the MIF^−/−^+TAA group (*p* < 0.001) (Figure 2). Further analysis of proinflammatory cytokines in myocardial tissue revealed that TNF levels were significantly higher in the TAA group (0.92 ± 0.04 pg/mg) compared to control values (0.23 ± 0.01 pg/mg) (*p* < 0.001). On the other hand, a significant decrease in myocardial TNF concentration was observed in the TAA+Bet (0.66 ± 0.07 pg/mg) and MIF^−/−^+TAA (0.82 ± 0.05 pg/mg) groups compared to the TAA group (*p* < 0.001 and *p* < 0.01, respectively). In addition, the TNF concentration in myocardial tissue was significantly reduced in the MIF^−/−^+TAA+Bet group (0.55 ± 0.09 pg/mg) compared to the TAA+Bet and MIF^−/−^+TAA groups (*p* < 0.01 and *p* < 0.001, respectively) (Figure 2). There were no significant differences in the concentration of IL-6 and TNF in the myocardial tissue between the MIF^−/−^ group and the wild-type mice fed the standard diet (Figure 2).

### 3.3. Profibrogenic Mediators (TGF-β1 and PDGF-BB) in Myocardial Tissue

**Figure 3:**
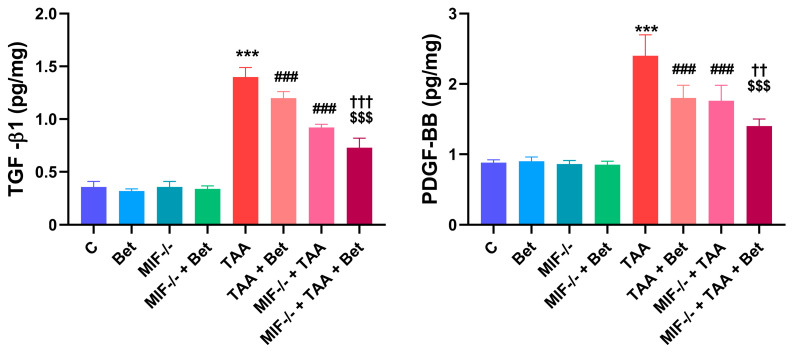
Effects of MIF and betain on the levels of profibrogenic mediators (TGF-β1 and PDGF-BB) in myocardial tissue of mice treated with TAA. The data are presented as mean ± SD from 7 mice per group. Values were normalized to the weight of myocardial tissue. Significance of the difference was estimated by using one-way analysis of variance (ANOVA) with Tukey’s post hoc test; *** *p* < 0.001 vs. C; ^###^ *p* < 0.001 vs. TAA; ^††^ *p* < 0.01 ^†††^ *p* < 0.001 vs. MIF^−/−^+TAA; ^$$$^ *p* < 0.001 vs. TAA+Bet.

There were no significant differences in the concentration of TGF-β1 and PDGF-BB in the myocardial tissue between the MIF^−/−^ group and the wild-type mice fed the standard diet (Figure 3). The TGF-β1 concentration in the myocardium was significantly increased (*p* < 0.001) in the TAA group (1.4 ± 0.09 pg/mg) compared to the control values (0.36 ± 0.05 pg/mg). The TGF-β1 concentration was reduced in the TAA+Bet (1.2 ± 0.06 pg/mg) and MIF^−/−^+TAA (0.92 ± 0.03 pg/mg) groups compared to the TAA group (*p* < 0.001). A significant decrease in TGF-β1 concentration was also observed in the MIF^−/−^+TAA+Bet group (0.73 ± 0.09 pg/mg) compared to the TAA+Bet and MIF^−/−^+TAA groups (*p* < 0.001). TAA treatment significantly increased the myocardial PDGF-BB concentration in the TAA group (2.4 ± 0.30 pg/mg) compared to the control (0.88 ± 0.04 pg/mg) (*p* < 0.001) (Figure 3). The myocardial PDGF-BB concentration was reduced in the TAA+Bet (1.8 ± 0.18 pg/mg) and MIF^−/−^+TAA (1.76 ± 0.22 pg/mg) groups compared to the TAA group (*p* < 0.001). In addition, the PDGF-BB concentration in the myocardium was significantly lower in the MIF^−/−^+TAA+Bet group (1.4 ± 0.1 pg/mg) compared to the TAA+Bet and MIF^−/−^+TAA groups (*p* < 0.001 and 0.01, respectively) (Figure 3).

### 3.4. Pathohistological Analysis of the Myocardial Tissue

**Figure 4:**
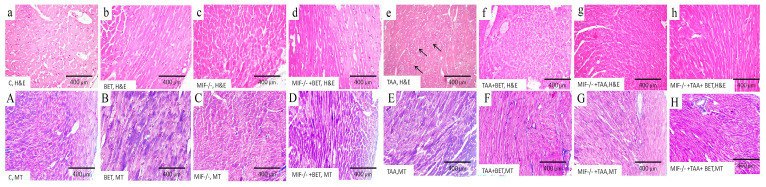
Histological appearance of myocardial tissue following TAA treatment. In the control groups (C (**a**), BET (**b**), MIF^−/−^ (**c**) and MIF^−/−^+BET (**d**)) myocardial tissue stained with H&E showed no morphological changes. MT staining of myocardial tissue in the control groups (C (**A**), BET (**B**), MIF^−/−^ (**C**) and MIF^−/−^+BET (**D**)) showed the absence of fibrosis. In the TAA-treated group, extensive fragmentation of cardiomyocytes was observed, with marked hypereosinophilia and nuclear loss (arrows) (**e**), without evidence of interstitial fibrosis (**E**). The TAA+Bet and MIF^−/−^+TAA groups exhibited moderate fragmentation of cardiomyocytes (**f**,**g**), without fibrosis on MT staining (**F**,**G**). Notably, the MIF^−/−^+TAA+Bet group showed significant histological improvement (**h**), comparable to the TAA+Bet and MIF^−/−^+TAA groups (**f**,**g**), also without fibrosis (**H**). Original magnification: ×400. Abbreviations: C—Control group; Bet—Betaine group; MIF—Macrophage Migration Inhibitory Factor; TAA—Thioacetamide; H&E—Hematoxylin and eosin; MT—Masson’s trichrome.

The myocardial tissue in the control groups (C and Bet) showed no morphological changes in the cardiomyocytes or the myocardial interstitium, confirming the absence of fibrosis on Masson’s trichrome (MT) staining (Figure 4). Moderate changes, such as eosinophilia of cardiomyocytes, were observed in the MIF^−/−^ group, while the MIF^−/−^+Bet group exhibited no significant alterations (Figure 4). In both groups, no interstitial fibrosis was observed on MT staining (Figure 4). In contrast, the TAA-treated group displayed extensive fragmentation of cardiomyocytes with hypereosinophilia and loss of nuclei (arrows), without evidence of interstitial fibrosis (Figure 4). TAA+Bet and MIF^−/−^+TAA showed moderate fragmentation of cardiomyocytes, without fibrosis on MT staining (Figure 4). The MIF^−/−^ group treated with both TAA and betaine (Figure 4) showed a significant histological improvement, comparable to the control group, also without fibrosis.

## 4. Discussion

Growing evidence indicates that CLD can lead to hemodynamic disturbances and consequently to heart failure through a complex interplay of different mechanisms such as systemic inflammatory mediators, oxidative stress, neurohumoral signals, vasoactive substances and others [56,57]. On the other hand, heart failure and CLD frequently co-occur due to etiological factors that impact both organs, such as alcohol abuse or drug toxicity [58]. Previously, our group had shown that betaine attenuated the proinflammatory and prooxidant effects of MIF in TAA-induced liver injury [9]. Furthermore, we have shown for the first time that MIF exhibits antifibrogenic activity in TAA-induced liver fibrosis and that betaine modulates this antifibrogenic effect of MIF [59]. The results of the present study show that MIF plays a significant role in the oxidative damage of myocardial tissue, as evidenced by a lower concentration of MDA, AOPP and nitrite in the knockout MIF^−/−^ mice treated with TAA compared to the TAA group. Consistent with this, MIF suppresses antioxidant myocardial defense in wild-type mice treated with TAA. MIF is produced by a wide range of cell types and is a ubiquitous protein found both inside and outside cells [60]. It has been proposed that MIF is a regulator of antioxidant response element (ARE) mediated gene transcription [60,61]. However, under inflammatory conditions, increased MIF signaling can be detrimental as it may trigger additional pathogenic signaling pathways [61]. MIF acts via both autocrine and paracrine mechanisms by binding to and activating multiple receptors, including CD74/CD44, CXCR2, CXCR4, and CXCR7. This receptor binding triggers several downstream signaling pathways in vivo, e.g., ERK1/2, AMPK, and AKT [62]. However, MIF signaling can vary depending on the tissue context. It is secreted by cardiomyocytes and can exert autocrine or paracrine effects. In cardiomyocytes, MIF expression is upregulated in response to oxidative stress. In addition, a decrease in endogenous MIF levels with age is considered to be a factor contributing to cardiac aging. In particular, MIF also plays a role in modulating the cardioprotective AMPK signaling pathway during ischemic events [63]. In this study, knockout MIF^−/−^ mice in TAA-induced liver fibrosis showed increased levels of SOD, CAT and thiols in myocardial tissue compared to wild-type mice. On the other hand, betaine successfully exerted its antioxidant effect in the myocardial tissue of wild-type mice treated with TAA and was even more successful in reducing oxidative damage in MIF^−/−^+TAA knockout mice, suggesting that betaine modulates the effects of MIF in myocardial tissue but may also exert an antioxidant effect through other mechanisms independent of MIF. Exposure to TAA leads to a significant increase in proinflammatory cytokines in heart tissue [64]. In CLD, tissue damage leads to the release of damage-associated molecular patterns (DAMPs), which favor a proinflammatory state. At the same time, portal hypertension and altered splanchnic hemodynamics contribute to increased intestinal permeability, allowing bacterial translocation and the entry of pathogen-associated molecular patterns (PAMPs) into the splanchnic, hepatic and systemic circulation. A key circulating proinflammatory cytokine in end-stage liver disease is TNF, which contributes to endothelial damage and promotes vascular permeability and vasodilation via a nitric oxide–mediated pathway [56]. It has also been shown that the concentration of circulating IL-6 correlates positively with the stage of liver cirrhosis [65]. Cardiomyocytes also produce IL-6 in response to injury [66]. Although IL-6 may play a cardioprotective role in cardiac tissue, chronically increased IL-6 signaling leads to maladaptive hypertrophy and impaired cardiomyocyte contractile function [67]. Our results showed that MIF was important for the synthesis of the proinflammatory cytokines IL-6 and TNF in the myocardium in a model of TAA-induced liver fibrosis. Several studies have demonstrated that the most severe toxic effects of TAA occur in the liver [8,13]. However, we cannot rule out the possibility that the observed myocardial tissue damage was, at least in part, due to the direct toxic effects of TAA. Betaine exhibited an anti-inflammatory effect by reducing the concentration of these cytokines in the myocardium of TAA-treated mice. This effect was even more pronounced in MIF deficiency. Although pathohistological analyses showed that no fibrosis occurred in the myocardial tissue in any of the experimental groups, an increase in the profibrogenic factors TGF-β1 and PDGF-BB was observed in the myocardial tissue of the mice treated with TAA. This finding indicates the presence of an early stage of cardiac tissue remodeling. Betaine administration reduced TGF-β1 and PDGF-BB levels in the myocardial tissue of wild-type mice treated with TAA, and this effect was even more pronounced in MIF^−/−^ knockout mice. This finding suggests that betaine achieves this effect by modulating MIF activity, but probably also by other mechanisms. Limitations of this study include the use of only male mice of a single age, the lack of functional assessments of the heart, and the evaluation of the role of MIF in myocardial oxidative stress and inflammation during TAA-induced liver injury at only one time point. Additionally, the observed oxidative stress and inflammation in myocardial tissue could, at least partially, be induced by TAA itself.

Liver cirrhosis is associated with impaired cardiovascular function and electrophysiological abnormalities. The impairment of cardiac function in patients with liver cirrhosis is caused, at least in part, by oxidative stress in cardiomyocytes and increased proinflammatory and profibrogenic mediators in myocardial tissue [56,57,64]. Our results indicate that in TAA-induced liver fibrosis, MIF plays a key mediating role in cardiotoxicity by promoting oxidative stress, inflammation, and fibrogenesis. On the other hand, betaine has the ability to exert not only antioxidant but also anti inflammatory and antifibrogenic effects in the myocardium either directly or via modulation of MIF activity in the coexistence of liver fibrosis. These results certainly argue in favor of conducting further research with betaine and determining a dose that has a beneficial effect on preventing the onset or progression of cardiac dysfunction in patients with liver cirrhosis.

## Data Availability

Data is contained within the article.

## Supplementary Materials

The following supporting information can be downloaded at https://www.mdpi.com/article/10.3390/cimb47090728/s1.

## Abbreviations

The following abbreviations are used in this manuscript:

MIF	Macrophage Inhibitory Factor
TAA	Thioacetamide
Bet	Betaine
IL-6	Interleukin-6
TNF	Tumor Necrosis Factor
TGF-β1	Transforming Growth Factor-beta1
PDGF-BB	Platelet Derived Growth Factor-BB
MDA	Malondialdehyde
AOPP	Advanced Oxidation Protein Product
SOD	Superoxide Dismutase
CLD	Chronic Liver Disease
HCC	Hepatocellular carcinoma
ECM	Extracellular Matrix
EDTA	Ethylenediaminetetraacetic acid
DTNB	2,2-dithiobis nitrobenzoic acid
PBS	Phosphate-buffered saline
NO_2_^−^	Nitrate
CAT	Catalase
GSH	Reduced Glutathione
H&E	Hematoxylin and Eosin
MT	Masson-Trichrome
ARE	Antioxidant response element
DAMPs	Damage-associated molecular patterns
PAMPs	Pathogen-associated molecular patterns
