# Supplementary material for: Macrophage Inhibitory Factor in Myocardial Oxidative Stress and Inflammation During Thioacetamide-Induced Liver Fibrosis: Modulation by Betaine
Source: Curr Issues Mol Biol. 2025 Sep 9;47(9):728. doi: 10.3390/cimb47090728 (PMC12468442; doi:10.3390/cimb47090728)
Supplement: Supplementary file 1 [file cimb-47-00728-s001.zip › cimb-3834281-supplementary.pdf]

Table S1. Body weight after the 8-week experimental period

Group	Body weight (g)
C	28.7 ± 2.1
Bet	26.1 ± 1.7
MIF$^{-/-}$	27.2 ± 1.9
MIF$^{-/-}$ + Bet	26.9 ± 2.2
TAA	23.8 ± 1.8***
TAA + Bet	25.9 ± 1.6
MIF$^{-/-}$ + TAA	25.1 ± 1.7*
MIF-/- + TAA + Bet	26.0 ± 1.6

Abbreviations: C, control group; MIF$^{-/-}$ group, mice knockout for macrophage migration inhibitory factor (MIF); Bet, betain group; MIF$^{-/-}$ +Bet group, knockout MIF mice who have received betaine; TAA group, animals who have received thioacetamide (TAA); MIF$^{-/-}$+TAA group, knockout MIF mice who have received thioacetamide; TAA+Bet group, animals who have received thioacetamide and betaine; MIF$^{-/-}$+TAA+Bet group, knockout MIF mice who have received thioacetamide and betaine. Significance of the difference was estimated by using one-way analysis of variance (ANOVA) with Tukey´s post hoc test; (*$p<0.05$ ***$p<0.001$ *vs.* C).
